# The Role of Platelet-Rich Plasma Injection for Muscle Strains in Athletes

**DOI:** 10.7759/cureus.60585

**Published:** 2024-05-19

**Authors:** David Vale, Adriana Pereira, José Paulo Andrade, João Paulo Castro

**Affiliations:** 1 Medical School, Faculty of Medicine, University of Porto, Porto, PRT; 2 Physical Medicine and Rehabilitation, Centro de Medicina de Reabilitação de Alcoitão, Alcoitão, PRT; 3 Unit of Anatomy, Department of Biomedicine, Faculty of Medicine, University of Porto, Porto, PRT

**Keywords:** platelet-rich plasma, return to play, muscles, return to sport, muscle tear, strains, athletes

## Abstract

Muscle tears/strains are among the most common musculoskeletal injuries, posing a serious challenge for sports medicine. Aiming to reduce the time to return to play and the rate of reinjuries, apart from the traditional conservative treatments and rehabilitation protocols, new and innovative therapeutic options have emerged, particularly platelet-rich plasma (PRP). This study aims to present the available evidence regarding PRP injection for the treatment of muscle strains in athletes.

Two databases were searched for articles published between January 2012 and December 2022 in Portuguese or English. The query used for the PubMed database was (“Muscles/injuries”[Mesh]) AND (“Athletes”[Mesh] OR “Athletic Injuries”[Mesh]) AND “Platelet-Rich Plasma”[Mesh], while for the Web of Science database the search was performed for “Platelet-rich plasma” AND “Muscle injuries” AND (“Athletes” OR “Athletic injuries”). Eleven studies involving athletes diagnosed with muscle injuries who received treatment with PRP injection alone, or in combination with traditional conservative treatment, compared to a control group, were included. Four randomized controlled trials, four systematic reviews/meta-analyses, two retrospective studies, and one comparative study were included.

Current evidence from the highest-quality studies does not support the hypothesis of reduction of time to return to play and the rate of reinjuries after PRP injection, even though some studies reported positive results. However, the available evidence suggests that PRP might have a beneficial effect on the pain perceived by athletes following an acute muscle strain.

It is challenging to arrive at definitive conclusions and translate these findings into a clinical context for treating muscle strains in athletes. The existing trials present several inconsistencies and limitations, with a heterogeneous set of patients and injuries, as well as the use of different and inconsistent methods for preparing, administering, and measuring the effects of PRP. To achieve consistent outcomes, standardizing PRP administration procedures is essential.

## Introduction and background

Muscle strains are among sports medicine’s most common musculoskeletal injuries [1-3]. They can be classified according to the trauma mechanism and typically occur in the distal myotendinous junction [4,5], occurring more frequently in biarticular muscles and in those mainly composed of type 2 fast-twitching muscle fibers [6-8]. They are reported with higher frequency in sports that require sprinting, kicking, acceleration, and rapid directional changes [9-11] and account for 37% of all time-loss injuries reported in professional soccer. Furthermore, injuries to the quadriceps femoris muscle and adductors, hamstrings, and calf muscle groups account for more than 90% of all muscle injuries in professional soccer [12].

Muscle injuries pose a genuine challenge for sports medicine due to the significant time lost for play and the elevated risk of reinjury [5,13]. In fact, one of the best predictors of muscle strain is a prior muscle injury [5,11,14-16]. Given this, despite the pressure to accelerate athletes’ recovery from muscle injuries, there is a great concern about preventing further lesions. In addition to conventional conservative treatments for muscle healing, there is a growing interest in novel and innovative therapeutic approaches that target minimizing the duration of return to play and the likelihood of relapse [13,17,18]. As such, ultrasound-guided injection of platelet-rich plasma (PRP) has emerged to boost the healing process after muscle strains [19,20].

The healing process is complex and dynamic and can be generally divided into the following three overlapping phases: inflammation, proliferation, and remodeling, with growth factors and cytokines organizing the normal evolution through these stages [1,21-23].

PRP is a biological autologous blood product characterized by a supraphysiologic platelet concentration [24,25]. It has anti-inflammatory and pro-regenerative properties, with platelets able to provide large amounts of cytokines and critical growth factors from their alpha granules, with a synergistic effect during the various stages of the healing process [1,21,26-28]. Numerous studies have reported its efficacy in enhancing the healing of tendons, ligaments, cartilage, and muscle [1,21-23,26,27].

Studies have highlighted several factors that may play a role in PRP efficacy, such as platelet concentration, activation of platelets, the presence of leucocytes [29,30], preparation volume [31], and pH differences [32]. Numerous extraction methods are available, each producing distinct PRP preparations with diverse compositions and presenting varying levels of efficacy [23,33,34].

Recently, PRP has been gaining attention as a minimally invasive treatment option. Numerous studies have examined its potential risks and benefits, both as a standalone treatment and in conjunction with physical rehabilitation. The goal of this study is to present a review of the available evidence regarding PRP injection in the treatment of muscle strains in athletes.

## Review

Methodology

Search Strategy

We searched the PubMed database using the query (“Muscles/injuries”[Mesh]) AND (“Athletes”[Mesh] OR “Athletic Injuries”[Mesh]) AND “Platelet-Rich Plasma”[Mesh]. Additionally, we also searched for articles through the Web of Science database using the query “Platelet-rich plasma” AND “Muscle injuries” AND (“Athletes” OR “Athletic injuries”).

Selection Criteria

A comprehensive search was conducted for randomized controlled trials (RCTs), systematic reviews, meta-analyses, and retrospective and comparative reviews published from January 2012 to December 2022. The inclusion criteria comprised articles involving athletes diagnosed with muscle injuries who received treatment with PRP injection alone or in combination with traditional conservative treatment and were compared to a control group (either placebo injection, conventional conservative treatment alone, or no therapy). The primary outcomes of interest were time to return to play, reinjury rate, and pain reduction. As for the exclusion criteria, we considered duplicated papers, narrative reviews, case reports, animal studies, non-muscle injury studies, study protocols, and incompatibility with the objective of this review.

Study Selection

The titles of the initially selected articles were independently analyzed by two investigators, who excluded any duplicates and those not deemed relevant to the research topic. The selection criteria were applied to the abstracts of the remaining literature, and a comprehensive examination of the complete papers was undertaken if needed.

Data Extraction

One investigator extracted data from the studies, with the second reviewer verifying the extracted information, which included the number of participants (for systematic reviews and meta-analysis, the number of trials was also obtained), type of intervention and control, duration of follow-up, and the results for the outcomes of interest.

Results

**Figure 1:**
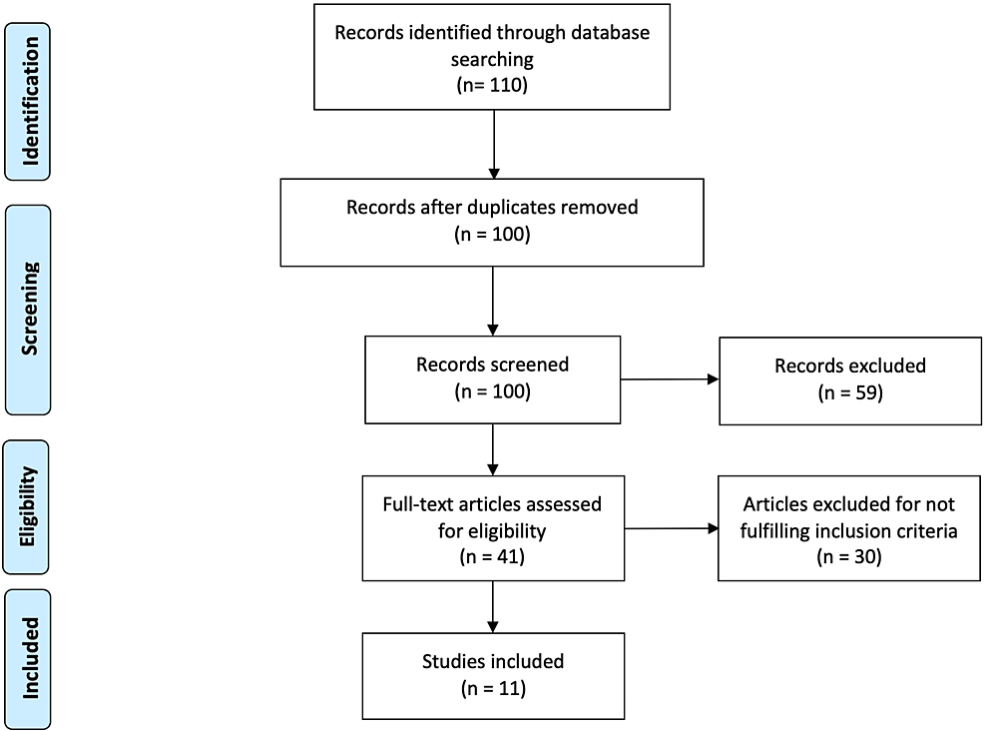
Preferred Reporting Items for Systematic Reviews and Meta-Analyses (PRISMA) flowchart of study selection.

This initial selection consisted of 110 articles. Ten articles were excluded because they were duplicates, whereas the complete texts were unavailable for 17 studies. After reading the titles and abstracts of the remaining 82 studies, only 11 met the selection criteria and were included in this review. These included four RCTs, four systematic reviews/meta-analyses, two retrospective studies, and one comparative study. The process of identification of relevant studies is described in Figure 1.

Randomized Controlled Trials

Hamilton et al. conducted a trial with 90 professional athletes who had MRI-positive hamstring injuries [35]. The participants were randomized into the following three treatment arms: injection with PRP intervention, injection with platelet-poor plasma (PPP), and no injection, with all participants being submitted to an intensive standardized rehabilitation program. The time to return to sport was significantly lower in the PRP injection group compared with the PPP group, with a difference of -5.7 days (95% confidence interval (CI) = -10.1 to -1.4; p =0.01). However, the difference was not statistically significant when comparing the PRP and the no injection arms, with a difference of -2.9 days (95% CI = -7.2 to 1.4; p =0.189), indicating that for athletes who had experienced acute hamstring injuries confirmed by MRI, the use of a single PRP injection did not offer any advantages in comparison to exclusively undergoing intensive rehabilitation. Differences between the groups regarding reinjury rate after two and six months were not reported [35].

In their study, Rossi et al. evaluated the duration required for recreational and competitive athletes to return to play and the likelihood of recurrence following acute grade 2 muscle injuries of the lower extremity (quadriceps femoris, gastrocnemius, or hamstrings) [36]. In a total of 75 athletes, the random allocation resulted in 34 patients receiving autologous PRP therapy plus physical rehabilitation, and 38 being submitted only to a rehabilitation program (control). The results indicated that the patients who received PRP therapy as part of their treatment protocol achieved full recovery significantly earlier than those in the control group (21.1 ± 3.1 days vs. 25 ± 2.8 days; p =0.001). Additionally, patients in the PRP group reported significantly lower levels of pain (evaluated using the Visual Analog Scale) throughout the study. However, no statistically significant difference was observed in the recurrence rate after a two-year follow-up period between the two arms of the study [36].

The study conducted by Reurink et al. aimed to study the potential acceleration of return to play following a hamstring injury through the application of PRP injections [37]. Eighty competitive and recreational athletes were randomly allocated to two groups. One protocol entailed administering two PRP injections (N = 41), with the first given within five days of the injury and the second administered five to seven days following the initial injection, while the other group received an isotonic saline injection (N = 39). According to the data obtained, intramuscular PRP injections did not provide any advantage in the time required for return to play over six months (hazard ratio = 0.96, 96% CI = 0.61 to 1.51), nor was there any benefit observed in the reinjury rate at two months or one year [37,38].

Hamid et al. (2014) conducted a single-blinded RCT to determine the effectiveness of a single autologous PRP injection in conjunction with a rehabilitation plan, versus a rehabilitation scheme alone, for the management of grade 2 hamstring injuries [39]. This study included a total of 28 patients, and the outcomes studied were time to return to play, with a significantly earlier full recovery in the PRP group (26.7 ± 7.0 days) compared with the control group (42.5 ± 20.6 days) (p = 0.02). The pain severity and pain interference scores were also assessed throughout the study by applying the Brief Pain Inventory-Short Form. The PRP protocol led to a statistically significant lower pain severity score, but no significant difference was found in the pain interference score, reflecting the impact of pain on daily activities [39].

Systematic Reviews and Meta-Analyses

In a 2021 meta-analysis executed to provide a formal presentation of the evidence regarding PRP injection as a treatment method for injuries to the hamstring muscles, Seow et al. included 10 studies, with a total of 356 patients (207 in the PRP group and 149 in the control group) [40]. Through the application of random and fixed-effects models, the authors concluded that the evidence analyzed favored the conjunction of PRP injection and physiotherapy, compared to physiotherapy alone or the absence of treatment, in a non-statistically significant way, regarding the occurrence of reinjuries (relative risk = 0.88, 95% CI = 0.45 to 1.71; p = 0.70) and the mean time to return to play (-5.67 days, 95% CI = -12.62 to 1.28; p = 0.11) [40].

Grassi et al. conducted a 2018 meta-analysis that analyzed data from six RCTs comparing PRP against at least one control group (placebo injection or physical therapy) for the management of acute muscle injuries, including foot and ankle, gastrocnemius, quadriceps femoris, rectus femoris, hamstrings, and shoulder [41]. Analyzing all six studies, with 374 participants, the time to return to sport was significantly reduced with the injection of PRP (-7.17 days, 95% CI = -12.26 to -2.08; p = 0.006). However, the risk of performance bias was high because there were only two double-blind studies, with four experiments lacking blinding of patients. If we consider only the double-blind studies, the variation found in the time to return to play is non-significant. Regarding imaging, muscle function, strength, pain, flexibility/range of motion, reinjuries, and complications, no notable differences existed between the PRP groups and the control group [41].

Reviewing five RCTs, comprising a total of 268 patients diagnosed with grade I or II acute (≤7 days) strains of the hamstring, thigh, foot, and ankle or shoulder muscles, in a 2018 meta-analysis, Sheth et al. concluded that, when compared to the control group (either placebo injection or physiotherapy), the participants that received PRP treatment achieved a statistically significant earlier return to sport, with a mean difference of -5.57 days (95% CI = -9.57 to -1.58; p = 0.006) [42]. Regarding the reinjury rate at six months of follow-up, no substantial variation was noted (odds ratio (OR) = 0.76, 95% CI = 0.34 to 1.69; p = 0.50) [42].

Regarding the efficacy of conservative interventions, such as PRP and rehabilitation (lengthening) exercises in acute hamstring injuries, Pas et al. conducted a meta-analysis in 2015 that encompassed RCTs comparing the effect of these conservative therapies in comparison to a control group or other interventions [43]. A total of 10 RCTs involving 526 patients were analyzed, and it was determined that these therapeutic measures had varying levels of efficacy. Specifically, the rehabilitation exercises were found to be the most effective. A meta-analysis of two studies revealed a statistically significant reduction in the time required for patients to return to play when compared to the control group (p < 0.0001). Conversely, the analysis of three studies investigating the use of PRP on acute hamstring injury revealed no significant difference in outcome when compared to the control group (p = 0.73) [43].

Retrospective Studies

In 2022, Trunz et al. analyzed the cases of 55 athletes who had sustained partial tears (grade 2 strains) of the hamstring muscles between 2013 and 2018 [44]. The authors aimed to compare the outcomes of a combination therapy consisting of PRP injection and hematoma aspiration with those of the traditional conservative treatment approach. The study population was matched in terms of age, the extent of muscle involvement, and the type of sport. The results indicated that the combination therapy had a beneficial effect, as it was associated with a significantly shorter time to return to play (23.5 ± 5.4 days vs. 32.4 ± 8.1 days; p < 0.001) and a lower incidence of recurrence (p = 0.025) [44].

In 2020, Bradley et al. performed a retrospective analysis on 69 National Football League players with MRI evidence of acute grade 2 hamstring injuries [45]. The study aimed to evaluate whether adding PRP injections to non-operative treatment would result in a shorter return to play. The study results revealed no statistically significant difference in the number of days missed (22.5 ± 20.1 days in the PRP group vs. 25.7 ± 20.6 days; p = 0.81) or the time to return to practice (p = 0.68) between the group receiving PRP injections and those not. However, the PRP group did have a faster return to play, with a difference of one game (1.3 ± 0.47 games vs. 2.9 ± 1.1 games; p < 0.05). Taking into consideration the possible financial repercussions of returning to play one game earlier, the utilization of PRP injections in professional athletes with acute grade 2 hamstring injuries may be considered beneficial [45].

Comparative Study

Aiming to compare the effects of PRP injection and conventional conservative treatment on pain and muscle function following acute muscle injury, Bubnov et al. conducted an experiment with 30 male professional athletes with an average age of 24 years [46]. The subjects were randomly divided into two groups. One group received ultrasound PRP injection and conservative treatment, and the other group received only conventional conservative treatment. All participants were evaluated on days 1, 7, 14, 21, and 28 following the initiation of treatment. The authors evaluated muscle function based on the range of motion, pain on resisted flexion, and strength, while the pain was evaluated using the Visual Analog Scale (0-10). Patients in the PRP group reported significantly greater pain relief on days 1, 7, 14, and 21 (p < 0.05), even though there was no significant variation at the conclusion of the observation period (28 days). Regarding muscle function, observations on the 7th and 14th days revealed significant differences (p < 0.05) in favor of the PRP group. However, on the 28th day, there was no substantial variation regarding muscle strength and pain during resisted flexion (p > 0.05). Despite this, the experimental group exhibited a significantly improved range of motion and subjective global function at the end of the observation period. The regenerative process, visualized on ultrasonography, was diagnosed earlier in group A, at 7 days after the start of treatment (p < 0.05), as well as on the 14th day of the protocol (p < 0.01). The regenerative process variation was not statistically significant on the evaluation after 21 days and onwards (p > 0.05). The average time for recovery and return to sport was reported to be 10 ± 1.2 days for group A and 22 ± 1.5 days for group B [46]. Table 1 summarizes the studies included in this review.

**Table 1:** Studies included in the review. RCT = randomized controlled trial; PRP = platelet-rich plasma; PPP = platelet-poor plasma; MRI = magnetic resonance imaging; RTP - return to play; ROM = range of motion; NFL = National Football League

Study	Summary	Results/Conclusion
Rossi et al., 2017 [36]	An RCT comparing PRP in addition to rehabilitation versus rehabilitation alone for the treatment of acute grade 2 lower extremity muscle injuries in 75 recreation and competitive athletes, with a two-year follow-up period	PRP promoted a shorter time to RTP after an acute grade 2 muscle injury, as well as significantly lower levels of pain. No significant difference was observed in the recurrence rate after a two-year follow-up period
Hamilton et al., 2015 [35]	A three-arm RCT comparing a single PRP injection following an MRI-positive hamstring injury in 90 professional athletes versus a PPP intervention or no injection, with all participants being submitted to an intensive standardized rehabilitation program	The PRP group demonstrated a significantly reduced time to return to sport compared with the PPP group, but the use of a single PRP injection was not advantageous when compared to the intensive rehabilitation-only group. No differences were reported regarding the reinjury rate after two and six months
Reurink et al., 2014 [37]	A multicenter, double-blind RCT evaluating double PRP injection effect on RTP after hamstring injury versus an isotonic saline solution injection in 80 recreational and competitive athletes	PRP did not provide any advantage in the time required for RTP over six months, nor was there any benefit observed in the reinjury rate at two months or one year
Hamid et al., 2014 [39]	A single-blind RCT comparing the effect of a single PRP injection plus rehabilitation program for the treatment of acute grade 2 hamstring injuries versus a rehabilitation scheme only in 28 patients	PRP provided a significantly earlier full recovery compared with the control group and a statistically significant lower pain severity score, but no significant difference was found in the pain interference score
Seow et al., 2021 [40]	A meta-analysis of 10 studies, with a total of 356 patients, to assess the effect of PRP and physiotherapy compared to physiotherapy-alone or no therapy for the treatment of hamstring injuries	The conjunction of PRP injection and physiotherapy in a non-statistically significant way reduced the occurrence of reinjuries and the mean time to RTP
Grassi et al., 2018 [41]	A meta-analysis of six RCTs, including 374 patients, comparing PRP therapy for the management of acute muscle injuries of the lower extremity and shoulder versus a control group receiving either a placebo injection or physical therapy	Time to RTP was significantly reduced with the injection of PRP (mean = 7.17 days). However, considering only the two blinded studies, this variation becomes non-significant. Pain, muscle function and strength, pain, imaging, flexibility/ROM, reinjuries, and complications, were not significantly different between the groups
Sheth et al., 2018 [42]	A meta-analysis of five RCTs, with a total of 268 participants diagnosed with grade I or II acute (≤7 days) muscle strains of the lower extremity and shoulder to compare PRP versus control (placebo injection or physiotherapy)	The PRP group achieved a statistically significant earlier return to sport, with a mean difference of -5.57 days (p = 0.006), but no substantial variation was noted in the reinjury rate at six months of follow-up (p = 0.50).
Pas et al., 2015 [43]	A meta-analysis of 10 RCTs, comprising a total of 526 patients, to compare the effects of PRP and rehabilitation exercises (not in conjunction) for the management of acute hamstring injuries	Rehabilitation exercises significantly reduced time to RTP when compared to the control group (p < 0.05). On the contrary, PRP demonstrated no difference in outcome when compared to the control group (p = 0.73)
Trunz et al., 2022 [44]	A retrospective analysis of 55 athletes who had sustained grade 2 hamstring strains between 2013 and 2018 to compare PRP plus hematoma aspiration versus the traditional conservative approach	The combination of PRP and hematoma aspiration led to a significant reduction in the time to RTP (p < 0.001) and in the incidence of recurrence (p = 0.025)
Bradley et al., 2020 [45]	A retrospective analysis of 69 NFL players with MRI-positive grade 2 hamstring injuries to assess the effects of the addition of PRP to conservative treatment	The PRP group returned to play one game earlier (p < 0.05), but there was no significant difference in the number of days missed (p = 0.81) or the time to return to practice (p = 0.68)
Bubnov et al., 2013 [46]	A comparative trial with a total of 30 male professional athletes comparing PRP injection and conventional conservative treatment versus conventional conservative treatment only	The PRP group reported significantly greater pain relief and muscle function during the study (p < 0.05) but not at the end of the observation period. ROM and subjective global function at the end of the observation period were significantly better in the PRP group, with ultrasound-visualized regenerative process diagnosed earlier in the PRP group

Discussion

Muscle injuries are one of the most common lesions in sports medicine. The sports and economic burden of time lost to play [5] has raised attention toward emerging autologous cellular therapies with PRP. The underlying scientific rationale behind PRP therapy is that the release of many biologically active factors from platelets, such as growth factors, cytokines, lysosomes, and adhesion proteins, support the three phases of wound healing (inflammation, proliferation, and remodeling) and might play a role in accelerating athletes’ recovery from muscle strains, as well as in the prevention of a recurrence [1,21-23,26,27].

Although several authors indicate that PRP injection reduces the time to return to play in muscle strains in athletes [36,39,42,44], the current evidence from the highest-quality studies does not support this hypothesis in a statistically significant way [35,38,40,41,43]. The reported recovery time after injury might be influenced by the heterogeneity of the rehabilitation protocol employed, as well as the absence of consensus about the optimal criteria for unrestricted activities.

Regarding pain reduction, an analysis of the available evidence reveals that PRP injection might have a beneficial role in lowering pain levels perceived by athletes after muscle strain [36,39,46].

Reinjuries are common, with imagological severity equal to or worse than the initial injury and a delayed return to sport [10]. Even though some authors suggest that PRP injection after muscle strains might decrease the risk of recurrence [44], the best-quality evidence fails to find any benefit in this regard [35,36,38,40-43].

The inconsistencies between different studies have challenged the applicability of PRP in clinical practice. The heterogeneity of the patients, types, and locations of muscle injuries, as well as the various timing and characteristics of PRP injection and the overall rehabilitation protocols, represent significant limitations regarding translating these results to clinical reality.

Although some studies have proposed different PRP classification systems to standardize their production, definition, and formulation, these attempts have been unsuccessful. Currently, the most used classification divides PRP into the following three groups: pure platelet-rich fibrin, leukocyte-rich PRP, and leukocyte-poor PRP [29,47-49]. Some studies argue that the lack of standardization of PRP preparation protocols has contributed to such clinical heterogeneity [50,51].

There is an ongoing debate regarding the best method for plasma centrifugation. While a single-step centrifugation protocol can increase platelet concentration by three times [52], a two-step method increases its concentration by eightfold and elevates the concentration of leukocytes [52]. However, a higher number of platelets within the preparation does not necessarily mean augmented healing due to a ceiling effect [22,32].

Some researchers argue that the timing of PRP injection is more important than the number of platelets injected [32]. The process of muscle repair involves the three overlapping phases already described, with growth factors and cytokines released by platelets organizing the normal evolution through these stages [1,21-23]. In PRP, these factors act synergistically, creating a microenvironment at the wound site by stimulating the formation of a fibrin matrix and the recruitment and differentiation of cells with a role in the healing process [1,21]. Pivotal growth factors consist of platelet-derived growth factor, vascular endothelial growth factor, transforming growth factor-beta (TGF-beta), basic fibroblast growth factor, epidermal growth factor, and insulin-like growth factor [1,21-23,53]. Although these substances might benefit the healing process [54], TGF-beta may have the opposite effect by inducting the development of fibrosis and inhibiting myogenic satellite cell proliferation and differentiation [55]. The activation of satellite cells in injured muscles leads to the proliferation and differentiation of myoblasts into skeletal myocytes and appears to be crucial in promoting adult skeletal muscle tissue regeneration following focal injuries [1]. There is ongoing research with promising results, indicating that the use of losartan (with potential systemic effects) [56,57] or neutralizing antibodies against TGF-b1 within PRP can enhance muscle regeneration and decrease collagen deposition (fibrosis) [57]. Although evidence suggests that PRP injection should be during or shortly after the inflammatory phase, there is a lack of evidence regarding the best therapeutic window. The exact location of the injections appears to be a critical factor in determining the effectiveness of PRP [32]. Factors such as the volume of PRP injected [31] and the presence of hematoma and its aspiration [44] may also impact the PRP effect, but the evidence is still lacking.

Regarding platelet activation, it can occur endogenously or before the preparation is injected via the incorporation of an exogenous clotting factor, such as thrombin or calcium chloride [29,47]. This activation induces degranulation, promoting the release of growth factors [21,29,47]. Although there is a lack of consensus about the best method, endogenous activation allows for a natural release pattern, resulting in a gradual release of growth factors as the platelets aggregate more slowly [47].

Leukocytes are pivotal in tissue healing due to their immune and host defense properties. While monocytes and macrophages participate in immunomodulation and tissue repair [58], lymphocytes produce insulin-like growth factors and support tissue remodeling [59]. The role of neutrophils in PRP is not yet understood. Even though a supranormal quantity of leukocytes can be deleterious [21], they are critical for the inflammatory response after muscle injury [60,61].

Despite the lack of evidence for the wide use of PRP injections in muscle strains in athletes, studies have confirmed its safety profile. As it is an autologous product, there are no known adverse effects [53], unlike other commonly used drugs in sports medicine, such as steroids [62].

## Conclusions

The evolution of healthcare is altering how medical procedures are performed, with minimally invasive techniques gradually taking over. PRP is emerging as a promising practice for sports medicine. Despite the encouraging biological foundation and positive results in preclinical studies, the shortage of literature and its limitations might hamper some outcomes from achieving statistical significance.

Meanwhile, the highest-quality studies suggest that PRP does not play a role in reducing the rate of reinjuries and shortening the time to return to play after muscle strains in athletes. On the other hand, the available evidence reports a beneficial effect on pain.

With this heterogeneous set of findings, it is challenging to arrive at definitive conclusions, highlighting the need for further research in this field. Several factors contribute to this, including different and inconsistent methods for preparing, administering, and measuring the effects of PRP and the diversity of musculoskeletal conditions treated.
